# Managing Acute Severe Ulcerative Colitis in 2025 and Beyond

**DOI:** 10.1007/s11894-025-01025-y

**Published:** 2026-01-07

**Authors:** Manjeet Kumar Goyal, Syed Adeel Hassan, Jeffrey Aaron Berinstein

**Affiliations:** 1 Department of Gastroenterology, All India Institute of Medical Sciences, New Delhi, India; 2 Department of Internal Medicine, Division of Gastroenterology and Hepatology, University of Michigan, 1500 East Medical Center Drive, Ann Arbor, MI USA

**Keywords:** Acute severe ulcerative colitis, Ulcerative colitis, Hospitalization, Infliximab rescue, Cyclosporine rescue, Upadacitinib rescue, Colectomy.

## Abstract

**Purpose of Review:**

Acute severe ulcerative colitis (ASUC) remains a high-risk condition with suboptimal clinical outcomes despite advancements in diagnostics, prognostication, and therapies. This review synthesizes recent evidence to address critical gaps in care, focusing on optimizing medical strategies to reduce colectomy rates and improve patient outcomes.

**Recent Findings:**

Recent studies have identified novel biomarkers and predictive models for stratifying patients as high-risk for colectomy. Several emerging therapeutic strategies to optimize care have also been explored. Intensified infliximab dosing has not consistently shown improved clinical outcomes across all patients with ASUC, though it may benefit a subset of patients with unfavorable pharmacokinetics. Furthermore, Janus kinase inhibitors have shown promise in reducing colectomy rates, offering a potential alternative for select patients; however, supporting evidence remains preliminary. Despite these advancements, colectomy remains exceedingly common but continues to serve as a critical intervention to reduce complications and mortality. This underscores the therapeutic efficacy ceiling that still exists in our current approach to ASUC in 2025.

**Summary:**

Modern ASUC management prioritizes rapid risk stratification (using clinical, endoscopic, and biomarker data) and patient-tailored advanced therapy selection. Future strategies should focus on conducting rigorous trials of emerging agents in comparison to our current protocols, while integrating real-time, personalized, and dynamic prognostic tools to reduce heterogeneity in treatment response.

## Introduction

Acute severe ulcerative colitis (ASUC) is a life-threatening presentation of ulcerative colitis (UC) that necessitates prompt initiation of inpatient medical treatment and assessment for surgical intervention [1, 2]. Despite substantial progress in diagnostic techniques, prognostication, and therapeutic options, the pace of advancement in managing ASUC has persistently lagged behind the rapid evolution observed in outpatient UC management [3]. ASUC management presents challenges despite these improvements, underscoring the need for further research and innovation. To date, first-line management remains anchored to intravenous (IV) corticosteroids— a 70-year-old intervention with unchanged unacceptably high colectomy rates (25–30%). Infliximab, which is the only biologic with proven efficacy as a rescue therapy, shows diminishing returns in biologic-refractory and extensively inflamed patients [4, 5]. While several prognostic scores are available, their clinical utility has been limited by poor sensitivity, specificity, and generalizability. This has resulted in a lack of formalized, dynamic, real-time decision thresholds on which to escalate care, leading to a reactive rather than proactive approach [6]. Recently, Janus kinase inhibitors (JAKi) have shown promise in reducing colectomy rates further [7, 8]. However, the optimal timing, sequencing, dosing, and positioning of these agents along with infliximab and cyclosporine remains to be established and warrant rigorous prospective study. Looking forward, the tailoring of these agents in a patient-specific manner, guided by individual pharmacokinetic and pharmacodynamic profiles, may be critical to overcoming the current therapeutic efficacy ceiling in ASUC. This article serves as an updated review of the management of ASUC in 2025 with a focus on recent developments, ongoing gaps in care, and insights into the future directions of ASUC management.

### Epidemiology and Disease Burden

ASUC, which is characterized by bloody diarrhea, tenesmus, urgency, abdominal pain, and systemic signs of inflammation, is associated with significant morbidity, mortality, and economic costs [1, 2, 9, 10]. 

Approximately 25–50% of UC patients will experience a severe flare during their disease course, with about 20% of UC patients presenting with a severe flare as their initial presentation [11–13]. A recent systematic review demonstrated stabilization of UC-related hospitalizations in developed countries, whereas UC-related hospitalizations appear to be rapidly increasing in newly industrialized countries [10, 14, 15]. 

In the pre-steroid era, mortality rates following an episode of ASUC were alarmingly high, reaching up to 40–75% within the first year [16, 17]. Fortunately, early initiation of IV corticosteroids, timely colectomy for steroid-refractory patients, and the integration of advanced therapies have lowered ASUC-related mortality to below 1% in the modern era [18–21]. However, mortality can increase to 5% in older patients or treatment-refractory patients who experience delays in colectomy [18–21]. 

UC-related hospitalizations impose a substantial economic burden, encompassing direct and indirect healthcare costs. Direct costs stem from prolonged inpatient care, advanced therapy administration, and surgical interventions. Indirect costs, while more challenging to quantify, are related to missed workdays, reduced productivity, and long-term physical and emotional disability [22–24]. 

Improved disease control through high utilization of biologic therapy was expected to reduce inpatient costs by decreasing hospitalization and surgery, however this has not been consistently observed [25]. In fact, the direct costs of UC-related hospitalizations have increased significantly over time. One recent study estimated an average cost for a non-surgical admission at $18,355 in 2017 (ranging from $5,771 to $28,069), which increased to $45,540 when surgery was required [14, 26]. With readmission rates as high as 20%, studies have shown that healthcare costs remain high following the initial admission for ASUC, further contributing to a sustained financial burden over time [27–29]. 

#### Pathogenesis

The pathogenesis of ASUC remains incompletely understood. ASUC is believed to share underlying mechanisms with UC, including the interplay of genetic predisposition, environmental triggers, and aberrant immune responses to gut dysbiosis. However, distinct genetic, microbial, and immunological profiles have been identified that correlate with more refractory disease courses [30]. These specific features provide insights into the heightened severity and treatment resistance characteristic of ASUC.

A genome-wide association study (GWAS) identified 46 single nucleotide polymorphisms, including the major histocompatibility region on chromosome 6p and the susceptibility gene *TNFSF15* (*TL1A*) on chromosome 9q, associated with medically refractory UC requiring colectomy [31]. More recently, a population-based GWAS analysis of 4491 patients demonstrated that carrying the HLA-DRB1*01:03 allele was associated with the need for surgery, hospitalization, and systemic corticosteroid use compared to noncarriers [32]. 

UC is associated with significant alterations in gut microbiota diversity, leading to changes in metabolomic and metagenomic profiles [33]. This dysbiosis, which is characterized by a reduction in beneficial organisms and an increase in pathogenic organisms, triggers a more aggressive immune response resulting in severe mucosal damage and ulceration [33, 34]. Specifically, reductions in beneficial taxa such as Ruminococcaceae, Faecalibacterium prausnitzii, and Prevotella and expansion of harmful bacteria such as Ruminococcus gnavus, Bacteroides fragilis, and Escherichia coli and have been observed in more severe UC, including ASUC [35]. These changes impair important anti-inflammatory processes, such as the production of short-chain fatty acids like butyrate, which are crucial for maintaining intestinal barrier function and regulating immune response [34, 36]. 

Intestinal epithelial barrier dysfunction plays a critical role in ASUC pathogenesis and progression. The intestinal epithelial barrier, which is made up of highly organized tight and adherens junctions, become compromised due to inflammation-induced alterations, leading to increased intestinal permeability and impaired barrier integrety [37]. Identifying reliable multiomic risk-factors and biomarkers could improve ASUC outcomes by enabling better patient risk stratification, in turn facilitating the timely initiation, escalation, and switching of effective therapies.

## Diagnostic Testing

Typically, patients are hospitalized for expedited work-up and treatment. Work-up should include obtaining vital signs and basic labs to assess for complications, exclude infections, quantify inflammation, and determine the safety for advanced therapy initiation. All patients should undergo testing for *C. difficile* infection, as *C. difficile* infection is associated with medication therapy failure, colectomy, and mortality [38, 39]. The presence of *C. difficile* infection should not delay medication initiation [40]. 

Flexible sigmoidoscopy should be performed within 48–72 h of admission to rule out cytomegalovirus (CMV) and determine the severity of inflammation. Early flexible sigmoidoscopy has been shown to reduce the rate of colectomy, length of admission, time to advanced therapy initiation, hospital costs, and mortality compared to sigmoidoscopy performed after 72 h [41–43]. Both CMV and Epstein-Barr virus (EBV) colitis have been associated with worse outcomes for ASUC, including poor response to medical therapy, colectomy, and prolonged hospital stay, and therefore should be excluded and treated if present [44–46]. Abdominal X-ray is used to rule out complications like perforation or toxic megacolon [47]. While obtaining cross-sectional imaging should be reserved for when there is concern for disease-related complications, emerging evidence suggests radiomic features of disease activity and nutritional status derived from colonic CT imaging may predict response to therapy and risk for colectomy [48]. 

Intestinal ultrasound (IUS) has emerged as a valuable, non-invasive tool for managing ASUC, by assessing initial disease activity as well as response to therapy. IUS can accurately assess colonic inflammation by measuring bowel wall thickness (BWT), vascularity, and wall stratification [49]. Several small studies have shown that baseline inflammation severity as measured by IUS within 24–48 h of IV corticosteroid initiation was predictive of non-responders to corticosteroids and the need for rescue therapy [50, 51]. A single-center study of 56 patients with ASUC demonstrated that a >20% reduction in BWT within the first 48 h predicted response to IV corticosteroids (odds ratio [OR] 22.6; 95% CI, 4.2–201.2.2.2) [52].

### Baseline Severity Assessment and Risk Factors for Colectomy

ASUC is defined by Truelove and Witts’ criteria, which includes the presence of more than six bloody stools per day along with any one of the following: heart rate >90 bpm, temperature >37.8 °C, hemoglobin < 10.5 g/dL, or erythrocyte sedimentation rate (ESR) >30 mm/h [2]. CRP >30 mg/L is often used in place of ESR, as CRP is more readily available and more responsive to changes in inflammation [2, 53]. The risk of colectomy increases with each additional criterion fulfilled, ranging from 8.5% (1 additional criterion) to 48% (≥ 3 additional criteria) [11]. The colectomy rate also increases with each subsequent UC-related admission - increasing from 19.9% (first admission) to 29.0% after two, 36.6% after three, and 38.2% after four admissions [11, 54, 55]. While Truelove and Witts criteria remain the most widely used criteria for identifying and defining ASUC, there are concerns that these criteria may not capture disease severity in our current era of biologics and advanced small molecules. Specifically, Truelove and Witts’ criteria may not accurately capture disease severity among patients who do not elevate systemic inflammatory markers or who may have an attenuated systemic response from their current incompletely effective outpatient therapies or chronic steroid use. While these patients may have a sub-acute presentation, they appear to be equally high-risk for colectomy [53, 56, 57]. 

Over the last 20 years, numerous baseline risk-factors of treatment non-response have been identified. These include frequent bowel movements, elevated CRP, low albumin, high fecal calprotectin (FCP), high CRP/albumin ratio, and severe endoscopic disease (specifically deep ulcers) [58–65]. Sarcopenia, current corticosteroids use, as well as prior thiopurine and anti-tumor necrosis factor (TNF) exposure have been identified as additional predictors of colectomy [58, 64]. 

Several additional scoring systems, incorporating a combination of the baseline risk-factors discussed above, have been developed to improve stratification and prognostication; however, the majority of these scoring systems are derived from small single-center cohorts and have not been prospectively validated [1, 53, 57, 61, 66, 67]. The ADMIT- ASC score was developed in the UK and externally validated in Australia and India. The score consists of CRP ≥ 100 mg/L (1 point), albumin ≤ 25 g/L (1 point), and a Ulcerative Colitis Endoscopic Index of Severity (UCEIS) score of 4–6 (1 point) or ≥ 7 (2 points) [68]. The ADMIT-ASC scoring was associated with corticosteroid response rates of 100% (0 points), 75.0% (1 point), 54.9% (2 points), 18.2% (3 points), and 0% (4 points) [68]. Table 1 provides a detailed overview of various baseline prognostic scores utilized in ASUC as well the components, strengths, and limitations of each score.

**Table 1:** Prognostic scoring systems in acute severe ulcerative colitis

Score (year)	When to apply	Variables	Outcome & key threshold(s)	Strengths	Limitations
Baseline (Admission) Scoring System					
Truelove & Witts’ Criteria (1955) [2]	Admission	≥ 6 bloody BMs/day and one of the following: - Temp > 37.8 °C (1 pt) - HR > 90 BPM (1 pt) - HgB < 10.5 g/dL (1pt) - ESR > 30 mm/h (1 pt)	6 Bloody BMs/day + 1 point = 8.5% 2 points = 31% ≥ 3 points = 48% risk for colectomy	- Simple, derived from readily available vital signs and labs - Widely used and validated - Used as the universally accepted definition of ASUC - Defines need for hospitalization - Useful for standardizing populations for clinical trials	- Developed in pre-advanced therapy era - Does not account for patients who do not mount systemic signs of inflammation or patients with suppressed systemic signs of inflammation due to corticosteroids - Not designed to predict colectomy or response to therapy - ESR is not as accurate at quantifying inflammation as CRP
The ACE (Albumin, CRP, and Endoscopy) Index (2021) [57]	Admission	CRP ≥ 50 mg/L (1 pt) Albumin ≤ 30 g/L (1 pt) Endoscopic Mayo = 3 (1 pt)	0 points = 12.9% 1 point = 31.5% 2 points = 45.5% 3 points = 78.1% risk for failing corticosteroids (need for rescue therapy or colectomy)	- Combines lab and endoscopic data for a more comprehensive assessment. - Modest predictive accuracy for corticosteroid failure	- Requires a recent endoscopy, which may not always be feasible or safe in ASUC - Requires external validation - Limited patients received advanced therapy in the derivation and validation cohort
Saint‑Antoine (Le Baut) index (2021) [39]	Admission	Prior anti-TNFs or thiopurine exposure (1 pt) Presence of CDI (1 pt) CRP > 30 mg/L (1 pt) Albumin < 30 g/L (1 pt)	0 points = 0.0% 1 point = 9.4% 2 points = 10.6% 3 points = 51.2% 4 points = 100% risk for colectomy within 1 year	- Prior exposure data, readily available baseline lab and concomitant infection data - Validated in geographically distinct populations - Useful for long‑term counseling	- Not beneficial for short-term outcomes - Limited patients received advanced therapy in the derivation and validation cohort
ADMIT-ASC Score (2023) [68]	Admission	Albumin < 25 g/L (1 pt) CRP > 100 mg/L (1 pt) UCEIS Score - 4–6 (1 pt) - ≥ 7 (2 pt)	0 points = 0% 1 point = 30% 2 points = 59% 3 points = 82% 4 points = 100% risk for failing corticosteroids (need for rescue therapy or colectomy)	- Combines lab and endoscopic data for a more comprehensive assessment. - High predictive accuracy for corticosteroid failure - Validated in geographically distinct populations - Provides early actionable triage for escalation and surgical intervention to minimize delays	- Requires a recent endoscopy, which may not always be feasible or safe in ASUC - Limited patients received advanced therapy in the derivation and validation cohort
Longitudinal (Following Admission) Scoring System					
Oxford (Travis) Index (1996) [69, 70]	Day 3 of IV corticosteroids	Non-response: (1) BMs > 8/day or (2) BMs 3–8/day + CRP > 45 mg/L.	Non-responders: 85% of patients required colectomy	- Simple to calculate using readily available data - High positive predictive value (PPV) for colectomy - Temporally validated - Comparison to other scoring systems show superior performance	- Small, single‑center derived prior to 1993 - Limited patients received advanced therapy limiting generalizability in the modern era - Not accurate for patients who do not mount CRP, have very high admission CRP, have high BM frequency at baseline, or had < 8 BMs/day on admission
Lindgren (Swedish) Score (1998) [71]	Day 3 of IV corticosteroids	Stool frequency/day + 0.14 × CRP (mg/L)	Score > 8 = 72% risk for colectomy	- Derived from readily available clinical and lab data	- Complex calculation - Small, single center cohort - Not validated - Limited patients received advanced therapy - Not widely used clinically
Ho (Edinburgh) Index (2004) [61]	Days 1–3 of IV corticosteroids	Colonic dilation > 5.5 cm (4 pt) Albumin < 30 g/L (1 pt) Average BMs over 3 BMs/day from Day 1–3 (Note < 4 BMs = 0 pt)	0–1 points = 11% 2–3 points = 45% ≥ 4 points = 85% risk for colectomy	- Simple to calculate using readily available data - Incorporates radiologic data - Temporally validated - Excellent discrimination between high and low risk patient	- Relies on abdominal X-ray, - Limited patients received advanced therapy - Relies heavily on colonic dilation (rare) which is a proximal marker for toxic megacolon and perforation and often an independent indication for surgery
All India Institute of Medical Sciences (AIIMS) Index (2017) [72, 73]	Day 3 of IV corticosteroids	Baseline UCEIS > 6 (1 pt) Day 3 FCP > 1,000 µg/g (1 pt)	AIIMS Score of 2 = 100% risk for steroid failure	- Combines lab and endoscopic data for a more comprehensive assessment - High PPV - Temporally and prospectively validated - Comparison to other scoring systems show superior performance	- Derived and validated in small single center cohort - Requires rapid FCP turnaround - Severity of ASUC questionable (moderate baseline FCP and CRP and low prior advanced therapy exposure) - Benefit of Day 3 FCP (over admission FCP) unclear
Dublin Index (Gibson CRP/Albumin Ratio) (2018) [60]	Day 3 of IV corticosteroids	CRP/albumin ratio (CAR) + > 3 BMs/day	CAR > 0.85 + > 3 BMs/day on Day 3 = likelihood ratio of 2.93 for failing corticosteroids	- Uses two objective markers of inflammation and nutritional status - Very simple to calculate	- Moderate PPV/NPV - Optimal cutoff threshold not universally established

Abbreviations: *AIIMS* All India Institute of Medical Science, *anti-TNF* Anti-Tumor Necrosis Factor, *ASUC* Acute Severe Ulcerative Colitis, *BM* Bowel Movement, *CAR* CRP/Albumin Ratio, *CDI* *Clostridioides difficile*; infection, *CRP* C-Reactive Protein, *ESR* Erythrocyte Sedimentation Rate, *FCP* Fecal Calprotectin, *HgB* Hemoglobin, *HR* Heart Rate, *NPV* Negative Predictive Value, *PPV* Positive Predictive Value, *pt* Point (s), *Temp* Temperature, *UCEIS* Ulcerative Colitis Endoscopic Index of Severity

### First-Line Therapy

#### Intravenous Corticosteroids

IV corticosteroids have been first-line for ASUC since 1955 [2, 74]. A landmark systematic review found that methylprednisolone 60 mg daily or hydrocortisone 100 mg.

every six hours for 3–7 days is the optimal dose for ASUC [64]. In our practice, we use methylprednisolone 30 mg twice daily to minimize nursing and patient administration burden while providing sustained plasma drug levels throughout the day.

#### Response Assessment To IV Corticosteroids

Response to corticosteroids is traditionally measured 72 hours after initiating IV corticosteroids using a combination of stool frequency and inflammatory biomarkers, most commonly by Oxford (Travis) criteria [69]. Meeting Oxford criteria, which is defined as having more than eight liquid bowel movements per day or having 3–8 bowel movements per day and a CRP >45 mg/L on Day 3, was associated with an 85%-risk of colectomy [69]. Derived from a small, single-center retrospective study in 1996, the Oxford criteria, may not adequately predict therapy response or colectomy in the modern era, especially in patients exposed to advanced therapies or those lacking elevation in systemic inflammatory markers [69, 72, 75]. Numerous unvalidated scoring systems have been developed (Table 1) over the years to assess response to corticosteroids from Day 3 onwards, most of which were derived from studies focusing solely on corticosteroid monotherapy therapy [60, 61, 71]. Recently, a scoring system developed at the All India Institute of Medical Sciences (AIIMS) consisting of baseline UCEIS ≥ 7 and Day 3 FCP >1000 µg/g was shown to have a specificity of 100% for predicting steroid non-response [73]. In a subsequent prospective validation study, the AIIMS index has been prospectively validated in a temporally distinct cohort at the same institution. This validation study showed that all patients meeting the AIIMS index criteria were classified as steroid non-responders (OR = 6.4; 95% CI = 2.1–80.2, *p* = 0.01), which outperformed the Oxford criteria (OR = 4.1; 95% CI = 1.2–78.4, *p*= 0.04 ) [72].

Regardless of the scoring system used, suboptimal response to 3 days of first-line IV steroids (or first-line advanced therapy) merits timely initiation of rescue therapy or referral for colectomy. Continuing high-dose corticosteroids beyond about 5–7 days without a satisfactory clinical response is generally discouraged, as prolonged ineffective medical therapy in ASUC is associated with higher rates of UC-related complications and mortality.

#### First-line Advanced Therapy

There remains a growing debate regarding whether initiation of advanced therapy should be delayed to Day 3 in order to assess corticosteroid response. Evidence is mounting that delayed initiation of rescue therapy for high-risk patients can lead to prolonged hospitalization, UC-related complications, colectomy, post-operative infections, and mortality [18, 20, 76, 77]. For this reason, our group has been advocating for upfront advanced therapy along with IV corticosteroids in patients determined to have a high baseline risk for failing corticosteroids or patients likely to require long-term advanced therapy maintenance. However, the problem remains on how to reliably identify patients at a high baseline risk for corticosteroid failure in order to justify aggressive early medical intervention.

### Second-Line Therapy

When IV corticosteroids fail to improve inflammatory burden by Day 3 of hospitalization, one must consider initiating second-line “rescue” therapies, which traditionally include IV cyclosporine, infliximab and, more recently, tofacitinib and upadacitinib. Each has distinct efficacy and safety profiles, and the choice of agent must be individualized based on individual patient factors, response, and comorbidities.

#### Intravenous Cyclosporine

Cyclosporine is a calcineurin inhibitor that was first shown in a 1994 landmark trial by Lichtiger et al. to induce an 82% (9/11) short-term clinical response in steroid-refractory patients (9/11 responders) compared to 0% (0/9) on placebo [78]. Current guidelines recommend administering IV cyclosporine at a dose of 2 mg/kg/day with target trough levels of 150–250 ng/mL. Dose selection was based on a subsequent trial showing comparable efficacy of IV cyclosporine 2 mg/kg/day compared with 4 mg/kg/day with less toxicity [79]. While cyclosporine has been shown to be an effective short-term rescue agent, its use is limited by a narrow therapeutic window, high nursing/physician burden, need for frequent monitoring, and high renal, neurologic, infectious, electrolyte, and hypertensive toxicities [80, 81]. In addition, intensive monitoring of drug levels and renal function is required throughout therapy adding further operational challenges. Moreover, cyclosporine is not suitable for maintenance due to high relapse rates and adverse events with long-term use [82]. Thus, if cyclosporine induces remission, it should be viewed as a bridge to a safer maintenance therapy (e.g. transitioning to thiopurines or biologic agents) rather than a sustained solution.

#### Infliximab

In a small randomized trial of 45 corticosteroid-refractory ASUC patients, Järnerot et al. found that a single infusion of infliximab 5 mg/kg improved 3-month colectomy rates compared to placebo [29% (7/24) vs. 67% (14/21), *p*= 0.017) [5]. While infliximab has a favorable safety profile with predictable dosing, the efficacy of infliximab is limited by several pharmacokinetic and pharmacodynamic factors [83]. Infliximab pharmacokinetics are impacted by high circulating levels of TNF-α, neutralization by anti-drug antibodies, reduced tissue drug penetration, low albumin and protein intake from malnutrition, colonic protein leakage, as well as colonic drug loss due severely damaged mucosal barrier from inflammation [83–86]. Escalated infliximab dosing strategies have been employed in an attempt to overcome high fecal drug clearance in patients with ASUC. Gibson et al. observed a lower 3-month colectomy rate using an accelerated infliximab regimen (defined as three doses of 5 mg/kg within 24 days) compared to standard dosing [6.7% (1/15) vs. 40% (14/35), *p*= 0.039] [87]. However it is important to note that there was no significant difference in steroid-free or colectomy-free survival long-term with accelerated or standard dosing strategies [88]. Despite initial promise with more aggressive infliximab dosing strategies, two systematic reviews and meta-analyses failed to demonstrate a difference between accelerated or 10 mg/kg (compared to standard 5 mg/kg) infliximab dosing for patients with ASUC [89–91]. 

PREDICT-UC, an open-label, multi-center, randomized trial, evaluated the efficacy and safety of intensified infliximab induction in steroid-refractory patients with ASUC. In this study, patients were initially randomized to receive infliximab 10 mg/kg (intensified induction [IIS]) or 5 mg/kg. Patients in the infliximab 5 mg/kg arm were re-randomized to standard induction (SIS) or accelerated induction (AIS). SIS patients received 5 mg/kg at weeks 0, 2, and 6, with an extra 5 mg/kg dose before day 7 if a patient did not respond, Whereas AIS patients received 5 mg/kg at weeks 0, 1 and 3, with the week 1 dose increased to 10 mg/kg and given earlier if a patient did not respond. The primary outcome (day 7 clinical response) was observed in 65% (30/46) of patients receiving 10 mg/kg compared to 61% (56/92) of patients receiving 5 mg/kg patients (*P*= 0.76) [92]. Following the second randomization, clinical response and colectomy-free survival was not significantly different between the three groups at 14 days, 3 months, or 12 months [92]. While the PREDICT-UC study showed no difference between standard, intensified, and accelerated induction strategies, non-responders were allowed to receive additional salvage infliximab doses, which potentially confounds the ability to detect differences between the initial dosing strategies. A follow-up pharmacokinetic analysis of patients enrolled in PREDICT-UC reported that lower serum infliximab concentrations and higher infliximab clearance on Day 3 were predictive of non-response and colectomy. They also found that administering higher initial or subsequent infliximab doses could counteract this high clearance [93]. These data suggest that individualized dosing strategies tailored to each patient’s response may be more effective than rigid, predetermined dosing schedules for patients with ASUC. The TITRATE Study used a pharmacokinetic-driven dashboard to tailor infliximab dosing for patients with ASUC [94]. In this study, patients were randomized to standard (SD) or personalized dosing of infliximab (PD), in which patients could receive additional infliximab infusions if a Bayesian pharmacokinetic algorithm predicted low infliximab serum concentrations. While the median cumulative infliximab dose by Day 42 was higher for the PD group (18.41 mg/kg vs. 13.79 mg/kg), the primary composite endpoint of clinical and endoscopic response at day 42 was not met (56.5% in PD vs. 44.0% in SD). However, by day 182 numerically more patients in PD achieved clinical remission (60.9% vs. 36.0%) and endoscopic remission (65.2% vs. 36.0%), although not statistically different [94]. These data suggest that the field is making progress in personalizing therapy. However, further research is necessary to integrate both baseline and dynamic multimodal models capable of identifying the most effective initial therapy and subsequently detecting suboptimal responses, facilitating timely escalation or switching to next-line therapies [95]. 

#### Comparative Effectiveness of Cyclosporine and Infliximab

Two head-to-head comparisons have demonstrated similar efficacy between cyclosporine and infliximab [4, 96]. The open-label CySIF study randomized 115 hospitalized steroid-refractory UC patients to receive either IV cyclosporine 2 mg/kg for 7 days (followed by oral cyclosporine for 98 days in addition to azathioprine) or infliximab 5 mg/kg at 0, 2, 6 weeks (in addition to azathioprine). No difference was observed in the 90-day rate of treatment failure, mucosal healing, AEs, or colectomy [infliximab: 21% (13/57) vs. cyclosporine: 17% (10/58), *p*= 0.60] [4]. Furthermore, long-term follow-up of patients treated in the CySIF trial failed to demonstrate a significant difference in colectomy-free survival at 1 year and 5 years; however, by 5 years, 62% of patients who received cyclosporine were on a new systemic therapy (mostly infliximab) compared to only 22% in the infliximab treatment arm [97]. Similar findings were seen in the larger CONSTRUCT trial, which showed no significant difference between IV cyclosporine 2 mg/kg for 7 days compared to standard-dose infliximab 5 mg/kg in regards to clinical efficacy, colectomy rates, or AEs [96]. A meta-analysis of non-randomized studies suggested comparable colectomy rates at 3 months, although infliximab was associated with a lower odds of colectomy at 1 year [98]. Despite similar short-term efficacy, clinicians often prefer infliximab for rescue therapy because of its superior safety profile, ease of use, lower monitoring and nursing requirements, and ability to continue infliximab long-term to maintain remission. Nevertheless, cyclosporine retains an important role for patients who are refractory to or have contraindications for infliximab, those with severe hypoalbuminemia, or when a rapid onset of action is required. Furthermore, its lower cost makes it a critical therapy in resource-constrained settings or where access to infliximab is limited.

#### Janus Kinase Inhibitors

Janus kinase inhibitors (JAKi), such as tofacitinib and upadacitinib, have recently emerged as promising therapeutic options for patients with ASUC. JAKi’s potent modulation of pro-inflammatory cytokines translates into several key clinical benefits, including predictable oral pharmacokinetics, rapid onset and clearance, comparable efficacy in biologic naïve patients and anti-TNF exposed patients, and the fact that JAKi are not susceptible to colonic drug loss associated with hypoalbuminemia and enhanced colonic permeability secondary to inflammation [99–103]. 

Tofacitinib, a pan-JAKi, and upadacitinib, a selective JAK-1 inhibitor, have been studied in many small case series of ASUC patients with good results [104–110]. A case-control study conducted at the University of Michigan compared 40 ASUC patients treated with upfront tofacitinib along with IV corticosteroids, matched 1:3 to usual care according to gender and date of admission. They found that tofacitinib was protective against colectomy at 90- days compared with usual care (hazard ratio [HR] 0.28, 95% CI 0.10–0.81). Interestingly, when stratified according to treatment dose, only the off-label high-intensity dose of 10 mg three times daily was protective (HR, 0.11; 95% CI, 0.02–0.56) against colectomy, whereas the FDA-approved dose of 10 mg twice daily was not (HR, 0.66; 95% CI, 0.21–2.09) [111]. In a recent systematic review consisting of 148 pooled ASUC cases, the authors found that tofacitinib use was associated with a colectomy-free survival of 85% at 30 days, 86% at 90 days, and 69% at 180 days, with 35% to 69% achieving clinical remission and 55% of patients achieving endoscopic remission [112]. Subsequently, the role of tofacitinib for patients with ASUC was evaluated prospectively in the TRIUMPH and TACOS study. The TRIUMPH study was an open-label single-arm prospective trial performed in Canada that treated 24 patients with ASUC refractory to three days of IV corticosteroids with tofacitinib 10 mg twice daily. Clinical response by Day 7, the primary outcome, was reached in 14 of 24 (58.3%) of tofacitinib-treated patients. At week 52, only 9/24 (37.5%) patients remained on tofacitinib, while 6 of 24 (25%) underwent colectomy by 90 days. The mean number of days to achieve clinical response was 2.4 reflecting tofacitinib’s rapid clinical onset. No new safety signals emerged in this acutely ill population. While only a small number of patients were enrolled in TRIUMPH, the results suggest that tofacitinib may be effective and safe for steroid-refractory patients within a week, although maintaining long-term remission may require additional optimization or subsequent therapy switches [8]. Furthermore, the largest prospective study evaluating the efficacy of tofacitinib for patients with ASUC was TACOS, a partially double-blind, randomized controlled trial conducted at an academic medical center in India. In TACOS, high-intensity, off-label tofacitinib (10 mg three times daily for 7 days) was used as an upfront indication strategy in combination with corticosteroids for patients with ASUC. In TACOS, 104 consecutive ASUC patients were assigned to either tofacitinib combined with IV hydrocortisone (100 mg every 6 h) or IV hydrocortisone alone (with matched placebo). By day 7, 83.0% (44/53) of patients in the tofacitinib arm achieved the primary endpoint— a decrease of more than three points in the Lichtiger index to a score below 10 on two consecutive days without rescue therapy—compared to 58.8% (30/51) in the corticosteroid monotherapy group (odds ratio [OR] 3.42, 95% CI 1.37–8.48, *p* = 0.007). Additionally, the 90-day need for rescue therapy (infliximab or colectomy) was 13% with tofacitinib compared to 38% with steroids alone (*p* = 0.003). Although most AEs were considered mild, one patient in the tofacitinib group developed a dural venous sinus thrombosis, and five patients died (1 in the tofacitinib group and 4 in the placebo group, of which two were post-colectomy) during the study period [7]. 

In a multi-center observational study conducted at four centers across the United States, 25 patients with ASUC were treated with upadacitinib combined with IV corticosteroids. Over 90 days, 24% of patients underwent colectomy, while 83% of patients avoiding colectomy achieved steroid-free clinical remission [104]. Similar results were observed when pooled in the systematic review [113, 114]. 

While JAKi has shown promise for treating patients with ASUC, several important questions remain unanswered regarding the timing of initiation, dosing, sequencing, and positioning of JAKi within ASUC management pathways. One ongoing trial (NCT05867329) and several planned trials of upadacitinib compared to IV corticosteroids or infliximab for patients with ASUC will hopefully provide additional data to address these critical gaps.

#### Emerging Therapies

Recent research explored the efficacy of interleukin-1 blockade with anakinra for ASUC. The multi-center IASO trial evaluating the interleukin-1 blocker anakinra for ASUC was stopped early for futility after an interim analysis showed numerically higher rates of medical rescue therapy (43% vs. 26%) and colectomy (11% vs. 4%) in the anakinra group compared to placebo [115, 116]. Hyperbaric oxygen therapy (HBOT) is being investigated due to its ability to reverse intestinal hypoxia, modulate inflammation by suppressing pro-inflammatory cytokines, and mobilize bone marrow-derived stem cells to promote mucosal healing [117]. A phase IIa pilot study of 18 patients hospitalized with UC (terminated early due to poor recruitment) compared daily HBOT with corticosteroids to sham hyperbaric air with corticosteroids. They observed higher rates of clinical remission (50 vs. 0%, *p* = 0.04) as well as lower rates of rescue therapy use (10 vs. 63%, *p* = 0.04) and colectomy (0 vs. 38%, *p* = 0.07) without any treatment-associated AEs in the HBOT group compared to sham [118]. In a phase IIb study, in which 20 patients hospitalized for a UC flare received 3–5 days of HBOT, 55% of patients experienced a reduction in clinical symptoms and inflammatory biomarkers at day 3, while only 15% (3/20) patients required rescue infliximab or colectomy [119]. A larger, multi-center, randomized, double-blind, sham-controlled trial is currently underway to confirm the observed benefits (NCT05987852).

### Third-line Therapy

#### Sequential Therapy

Combining advanced therapies has emerged as an option for UC patients unable to achieve a complete response or remission to advanced monotherapy [120, 121]. Sequential rescue therapy has been used for steroid-refractory ASUC patients who have failed corticosteroids and initial rescue therapy with mixed results. A meta-analysis including 23 studies and a total of 340 patients with ASUC, observed that sequential rescue therapy with infliximab and cyclosporine (or vice versa) was associated with a colectomy-free survival of 53%, an adverse event rate of 26%, and a mortality rate of 0.88% (3 deaths) [122]. On account of the high mortality and adverse event rate, most guidelines and protocols recommend against the use of infliximab after cyclosporine or vice versa. However, more recently several case series have explored the use of tofacitinib and upadacitinib after infliximab or cyclosporine rescue therapy and have shown encouraging rates of colectomy avoidance and infections [123–126]. Our group conducted a multi-center retrospective review of 21 patients with ASUC who received infliximab and either tofacitinib or upadacitinib during the same admission. Over 16 weeks, the colectomy rate was 38% (8/21) and the steroid-free clinical remission was 91% among patients avoiding colectomy. Only four patients developed an AE, most of which were not classified as severe [127]. It is important to highlight that colectomy would have been recommended to all these patients due to insufficient response to corticosteroids and initial advanced therapy; However, 62% avoided colectomy without significantly increasing AEs. However, it is important to note that the evidence supporting sequential JAK inhibitor rescue therapy after infliximab or cyclosporine is limited to small case series and cannot be recommended or generalized. Accurate identification of ASUC patients likely to fail initial advanced therapy failure is critical to determine who may benefit from sequential advanced therapy. Pre-treatment variables such as high CRP, low serum albumin, baseline calculated infliximab drug clearance, and elevated band neutrophil count at the time of infliximab administration have been shown to predict suboptimal infliximab response and subsequent need for colectomy [67, 128–132]. Following infliximab administration, absolute CRP and percent CRP reduction, CRP to lymphocyte ratio, CRP to albumin ratio, serum and fecal infliximab concentration, and cytokine profile have been shown to predict sub-optimal infliximab response, reflecting both ongoing systemic inflammation and impaired drug pharmacokinetics [93, 130, 132–134]. 

### Surgical Management

Despite substantial advances in the medical management for ASUC, colectomy remains a critical intervention for patients who develop life-threatening complications or fail to respond to medical management [135]. In the acute setting, the preferred surgical procedure is a subtotal or total abdominal colectomy with end ileostomy, which leaves the rectal stump in place [136]. This approach is favored over immediate total proctocolectomy because it is technically less demanding, reduces operative time, and is associated with lower rates of pelvic sepsis and anastomotic complications, particularly in patients who are acutely ill, immunosuppressed, or nutritionally depleted [137–139]. Following recovery from the ASUC episode and the initial operation, patients may elect to undergo restorative proctocolectomy with ileal pouch-anal anastomosis (IPAA). This is most commonly performed as a three-stage approach according to the following sequence: (1) the initial subtotal colectomy and end ileostomy; (2) completion proctectomy and IPAA creation with a diverting loop ileostomy; and (3) reversal of the loop ileostomy [136]. 

#### Indications and Timing for Surgery

The indications for surgery in the management of ASUC are well-defined. Absolute indications for urgent colectomy include toxic megacolon, colonic perforation, pelvic sepsis, peritonitis, intractable hemorrhage, and shock [135, 136]. Surgery should also be considered in patients who fail to demonstrate substantial clinical improvement after first-line intravenous corticosteroids and/or second-line rescue therapy, as delays in operative intervention are associated with increased postoperative morbidity and mortality [18, 20, 140, 141]. Consensus statements and guidelines recommend surgical consultation for hospitalized patients with ASUC within 72 h of therapy for all patients without a robust improvement [136, 142]. However, the optimal timing of surgery for patients with medically refractory disease is more nuanced and requires careful, ongoing assessment. The initial benchmark is a lack of clinical and biochemical response after 3–5 days of first-line IV corticosteroids as outlined according to Oxford criteria (See: First-Line Therapy: *Response Assessment to IV Corticosteroids*). However, for patients who receive infliximab, cyclosporine, tofacitinib, or upadacitinib, there is no consensus on the optimal criteria or time point to define rescue therapy failure. Clinicians must rely on a variety of parameters, including persistent symptoms and inflammatory markers or lack of improvement on intestinal ultrasound or endoscopy. This absence of standardized and validated criteria contributes to significant practice variability and likely contributes to suboptimal outcomes related to delaying necessary surgery. Given that patients can deteriorate unpredictably and develop life-threatening complications at any time during hospitalization, we advocate for close clinical and biochemical monitoring with early colorectal surgeon consult within 24–48 h of admission for any patient with ASUC. This early involvement is essential to establishing a collaborative approach, educating the patient and family on the potential need for surgery, and to facilitate a timely and efficient transition to the operating room if and when it becomes clear that complications are developing or medical management has failed [136, 142]. 

#### Optimizing Surgical Outcomes

Optimizing surgical outcomes in ASUC requires a multidisciplinary approach that addresses modifiable risk factors. Aside from a standard preoperative history and work-up, special attention should be paid to the dose, timing, and duration of corticosteroids and other therapies as well as weight loss, exercise tolerance, functional status, prior abdominal surgery, obstetrical history, family planning goals, prior pelvic radiation, and history of incontinence [143]. Establish risk factors for adverse perioperative and postoperative outcomes includes preoperative high-dose corticosteroids, malnutrition, anemia, and delayed surgery or early pelvic dissection [18–20, 140, 141, 144–146]. Meanwhile, immunomodulators (6-mercaptupurine, azathioprine, and methotrexate), anti-TNF therapy, and tofacitinib have not definitively been associated with increased perioperative and preoperative complciations [144, 147–151]. While early studies showed an association between preoperative exposure to anti-TNF therapy and anastomotic leak and pelvic sepsis, this was not seen several subsequent studies including the PUCCINI (Prospective Cohort of Ulcerative Colitis and Crohn’s Disease Patients Undergoing Surgery to Identify Risk Factors for Post-Operative INfection I) trial, largest prospective study to date [144, 151–154]. Specifically, in the context of ASUC, we participated a multi-center, retrospective, case-control study of hospitalized patients who underwent colectomy, comparing 41 patients who were treated with tofacitinib to 68 treated with infliximab before colectomy [150]. Compared to tofacitinib-treated patients, infliximab-treated patients had higher rates of overall (44 [64.7%] vs. 13 [31.7%], *p* = 0.002) and severe (19 [27.9%] vs. 3 [12%], *p* = 0.019) post-operative complications however no significant difference was observed in the risk for developing serious post-operative complications (OR 0.28 [95% CI 0.06, 0.96]; *p* = 0.61) on multivariable analysis [150]. Although small, these results provide reassurance for providers that to use of JAKi with caution in hospitalized patients with ASUC due to potential concerns for major cardiovascular events, venous thromboembolism, and severe infections These results are consistent with broader evidence that suggests the use of advanced therapy in combination with corticosteroids for acutely-ill hospitalized patients likely does not increase perioperative risk. As a result, guidelines and consensus statements recommend that the decision to perform colectomy should not be delayed or influenced by prior medication exposure [136]. Most hospitalized patients with UC undergoing surgery will follow a standard enhanced recovery pathway which includes multimodal pain management, early ambulation, and venous thromboembolic prophylaxis for 30 day post-operatively. Systemic therapy including corticosteroids should be tapered off according to the dose and duration of pre-colectomy exposure. Generally, the second stage of restorative proctocolectomy (completion proctectomy with IPAA) should be delayed until corticosteroids have been weaned, disease is controlled, and the patient’s nutritional status is optimized. During this time, it is critical to reassess for features suggestive of Crohn’s disease, as the presence of Crohn’s disease is a contraindication to IPAA due to high rates of pouch failure [155, 156]. This assessment should include a thorough review of available pathology, small bowel endoscopy, and cross-sectional imaging for any features suggestive of pre-operative Crohn’s (i.e., small bowel involvement, perianal disease, or granulomas) especially where initial diagnosis is indeterminant.

### Long-Term Prognosis

Despite considerable advancements, colectomy rates remain clinically significant. While reported outcomes vary across studies, contemporary data consistently demonstrate a 90-day colectomy rate of at least 20–30%, rising to 30–40% within one year of index hospitalization [4, 11, 58, 80, 97, 157]. The cumulative risk of colectomy continues to climb over time and with subsequent admissions [11, 54, 55]. This underscores the need to focus on post-discharge management, as nearly equal numbers of patients undergo colectomy after hospitalization as during initial hospitalization [29]. 

Studies have consistently shown that patients who clear their inflammatory burden without the use of corticosteroids are less likely to require delayed colectomy [158, 159]. To achieve this, advanced therapy initiation should be prioritized during admission or shortly after discharge for ASUC patients likely to require long-term advanced therapy maintenance. Two recent large studies demonstrated that corticosteroid-responsive patients discharged on mesalamine experienced higher rates of disease progression (ongoing steroid use, therapy change, hospitalization, or colectomy) compared to patients initiated on immunomodulators or advanced therapy [160, 161]. 

### Future Directions and Unmet Needs

The management of ASUC has evolved tremendously since it was first described over 70 years ago. The incorporation of IV corticosteroids, novel advanced therapies, and early surgical intervention have substantially reduced the overall mortality from over 40% to less than 1% [2, 16, 21]. This remarkable improvement has shifted the focus of clinical outcomes towards improving colectomy rates, which remain unacceptably high. Despite significant medical advances, ASUC continues to represent a serious clinical challenge, with 30%−40% of patients still requiring colectomy [4, 11, 58, 80, 97, 157]. This persistent issue underscores that fundamental knowledge and management gaps need to be addressed to improve ASUC outcomes.

These gaps include a better understanding of the disease pathophysiology, more accurate predictors of treatment response, optimization of current therapies, and development of novel targeted interventions that are studied through rigorous controlled trials [3, 162]. Additionally, improved risk stratification tools are needed to identify patients at high risk of colectomy early in their disease course, allowing for more aggressive and timely interventions.

Emerging data suggest that novel, rapid-onset JAK inhibitors have an important role in ASUC treatment pathways [7, 104]. However, prospective studies are needed to define optimal positioning, sequencing, and dosing relative to IV corticosteroids, infliximab, and cyclosporine.

Even with the incorporation of these novel therapeutic agents, a therapeutic ceiling remains— highlighting the potential value of sequencing or combining advanced therapies. The integration of novel, fast-acting, and rapidly cleared agents such as JAKi may make sequential or combination approaches reasonable in select patients.

Nonetheless, pharmacologic innovation alone is insufficient. The substantial heterogeneity in patient pathophysiology and pharmacokinetics highlights the critical need for personalized treatment strategies. These strategies must go beyond the utilization of static baseline clinical risk factors to the integration of multimodal, dynamic, and adaptive models that evolve throughout a patient’s clinical course—enabling timely escalation or switching of therapy based on predicted response trajectories.

Equally important is the recognition of the psychological toll of hospitalization in ASUC. With up to 40% of patients identifying IBD-related hospitalization and poorly controlled pain and anxiety during that hospitalization as the source of their medical post-traumatic stress— highlighting a pressing need to design patient-centered interventions that not only optimize medical care but also address the psychological trauma associated with severe disease flares [24, 163–165]. 

Despite these challenges, the remarkable progress achieved so far inspires optimism for the future. As research continues to expand our understanding of disease mechanisms and optimal therapeutic pathways, the future of ASUC care appears bright, driven by a commitment to innovative, personalized, and patient-centered approaches to care.

## Key References

- Adams A, Gupta V, Mohsen W, Chapman TP, Subhaharan D, Kakkadasam Ramaswamy P, et al. Early management of acute severe UC in the biologics era: development and international validation of a prognostic clinical index to predict steroid response. Gut. 2022 Sep 28;gutjnl-2022-327533.
  - This manuscript presents a predictive model developed in the Oxford UK (and validated in Australia and India) to identify patients at high risk for requiring medical rescue therapy or colectomy. The model consisted of CRP of ≥ 100 mg/L (one point), albumin of ≤25 g/L (one point), and UCEIS score of ≥4 (1 point) or ≥7 (2 points). Patients scoring 0, 1, 2, 3 and 4 in the validation cohorts had steroid response rates of 100%, 75.0%, 54.9%, 18.2% and 0%, respectively. Therefore, the authors proposed upfront rescue therapy or referral for colectomy for patients with a baseline score of ≥3 points.
- Honap S, Jairath V, Sands BE, Dulai PS, Danese S, Peyrin-Biroulet L. Acute severe ulcerative colitis trials: the past, the present and the future. *Gut*. Sep 09 2024;73(10):1763-1773. 10.1136/gutjnl-2024-332489.
  - This article systematically reviews randomized controlled trials conducted in patients hospitalized with UC, highlighting key advancements in treatment from cortisone in 1955 to cyclosporin in the 1990s, infliximab in the early 2000s, and tofacitinib in the 2020s and early 2000s. It examines the evolution of trial designs, eligibility criteria, and study populations while identifying gaps in drug development. The review also discusses ongoing trials and proposes strategies to overcome challenges in future clinical programs, making it a valuable resource for understanding ASUC treatment progress in order to guide future research.
- Kayal M, Meringer H, Martin L, Colombel JF. Systematic review: Scores used to predict outcomes in acute severe ulcerative colitis. Aliment Pharmacol Ther. Nov 2023;58(10):974-983. https://doi.org/10.1111/apt.17731
  - This systematic evaluates 19 predictive scores for outcomes in ASUC, including intravenous corticosteroid response and colectomy risk. The article categorizes the different scoring systems based on factors such as complexity, the variables assessed, and the time points at which they are applied.
- Choy MC, Li Wai Suen CFD, Con D, et al. Intensified versus standard dose infliximab induction therapy for steroid-refractory acute severe ulcerative colitis (PREDICT-UC): an open-label, multicentre, randomised controlled trial. Lancet Gastroenterol Hepatol. Nov 2024;9(11):981-996. 10.1016/S2468-1253(24)00200-0.
  - This is the first randomized trial to investigate the efficacy of different infliximab dosing strategies for patients with steroid-refractory ASUC. The study found no significant difference in clinical response rates by day 7 between patients receiving a higher dose of 10 mg/kg and those receiving a standard dose of 5 mg/kg of infliximab (65% vs. 61%). Additionally, the trial demonstrated that intensified and accelerated dosing regimens did not lead to better outcomes compared to standard induction therapy up to three months later, concluding that infliximab is a safe and effective rescue therapy for ASUC, but escalating the initial dose may not provide additional benefits.
- Singh A, Goyal MK, Midha V, et al. Tofacitinib in Acute Severe Ulcerative Colitis (TACOS): A Randomized Controlled Trial. Am J Gastroenterol. Jul 01 2024;119(7):1365-1372. 10.14309/ajg.0000000000002635.
  - TACOS which is a single-center randomized controlled trial, examines the efficacy of tofacitinib 10 mg three times per day combined with IV corticosteroids compared to IV corticosteroids alone in treating hospitalized with ASUC. The trial found that 83% of patients receiving tofacitinib combined with IV corticosteroids met the primary outcome, day 7 response, compared to 58% in the IV corticosteroids monotherapy group. indicating a significant improvement in treatment responsiveness. The study suggests that tofacitinib 10 mg three times per day combined with IV corticosteroids is more effective than IV corticosteroids alone in producing an early response without rescue therapy or colectomy.

## Data Availability

No datasets were generated or analysed during the current study.
